# Optimized type-2 fuzzy controller based on IoMT for stabilizing the glucose level in type-1 diabetic patients

**DOI:** 10.1038/s41598-023-41522-6

**Published:** 2023-09-04

**Authors:** Amged Sayed, Belal A. Zalam, Mohanad Elhoushy, Essam Nabil

**Affiliations:** 1 Department of Industrial Electronics and Control Engineering, Faculty of Electronic Engineering, Menoufia University, Menouf, 32952 Egypt; 2 Department of Electrical Energy Engineering, College of Engineering and Technology, Arab Academy for Science Technology and Maritime Transport, Smart Village Campus, Giza, Egypt

**Keywords:** Computational biology and bioinformatics, Engineering, Biomedical engineering, Electrical and electronic engineering

## Abstract

Due to advancements in existing Internet of Medical Things (IoMT) systems and devices, the blood glucose level (BGL) for type-1 diabetic patients (T1DPs) is effectively and continually monitored and controlled by Artificial Pancreas. Because the regulation of BGL is a very complex process, many efforts have been conducted to design a powerful and effective controller for the exogenous insulin infusion system. The main objective of this study is to propose an optimized interval type-2 fuzzy (IT2F) based controller of artificial pancreas for regulation BGL of T1DP based on IoMT. The proposed controller should avoid the risk of hyperglycemia and hypoglycemia situations that T1DP faces during the infusion of exogenous insulin. The main contribution of this work is using meta-heuristic method called grey wolf optimizer (GWO) to tune the footprint of uncertainty for IT2F’s membership functions to inject the proper dose of insulin under different conditions. The nonlinear extended Bergman minimal model (EBMM) with uncertainty is used to represent the blood glucose regulation and represent the dynamics of meal disturbance in T1DP. The effectiveness and the performance of the proposed controller are investigated using MATLAB/Simulink platform. Simulation results show that the proposed controller can avoid both severe hypoglycemia and hyperglycemia for nominal parameters of the model, in addition to model under the presence of both parametric uncertainty and uncertain meal disturbance.

## Introduction

Since the discovering of insulin by Frederick G Banting, Charles H Best and JJR Macleod, many efforts have been conducted sequentially to regulate of blood glucose level (BGL) for type-1 diabetic patients (T1DPs). BGL in a human body must be naturally within the physiological range of 70–180 mg/dl^1,2^. Regulation of BGL is a very complex process that comes through the interaction of several feedback mechanisms of many vital organs such as liver, brain, intestines, pancreas, and others^3^. The traditional therapy (open loop control method) of treating patients with type-1 diabetic mellitus (T1DM) is based on measuring the BGL of patient several times daily using blood glucose meter and if the BGL is at high values, then according to the values of the measurements the right dose of exogenous insulin is calculated and then it is infused subcutaneously into patient to lower the BGL^4^. But this method is painful, unreliable, time consuming, and not suitable at night during sleep hours^4^. Because of these shortcomings of the traditional method, a new technology called artificial pancreas (AP) has been developed^5^.

**Figure 1:**
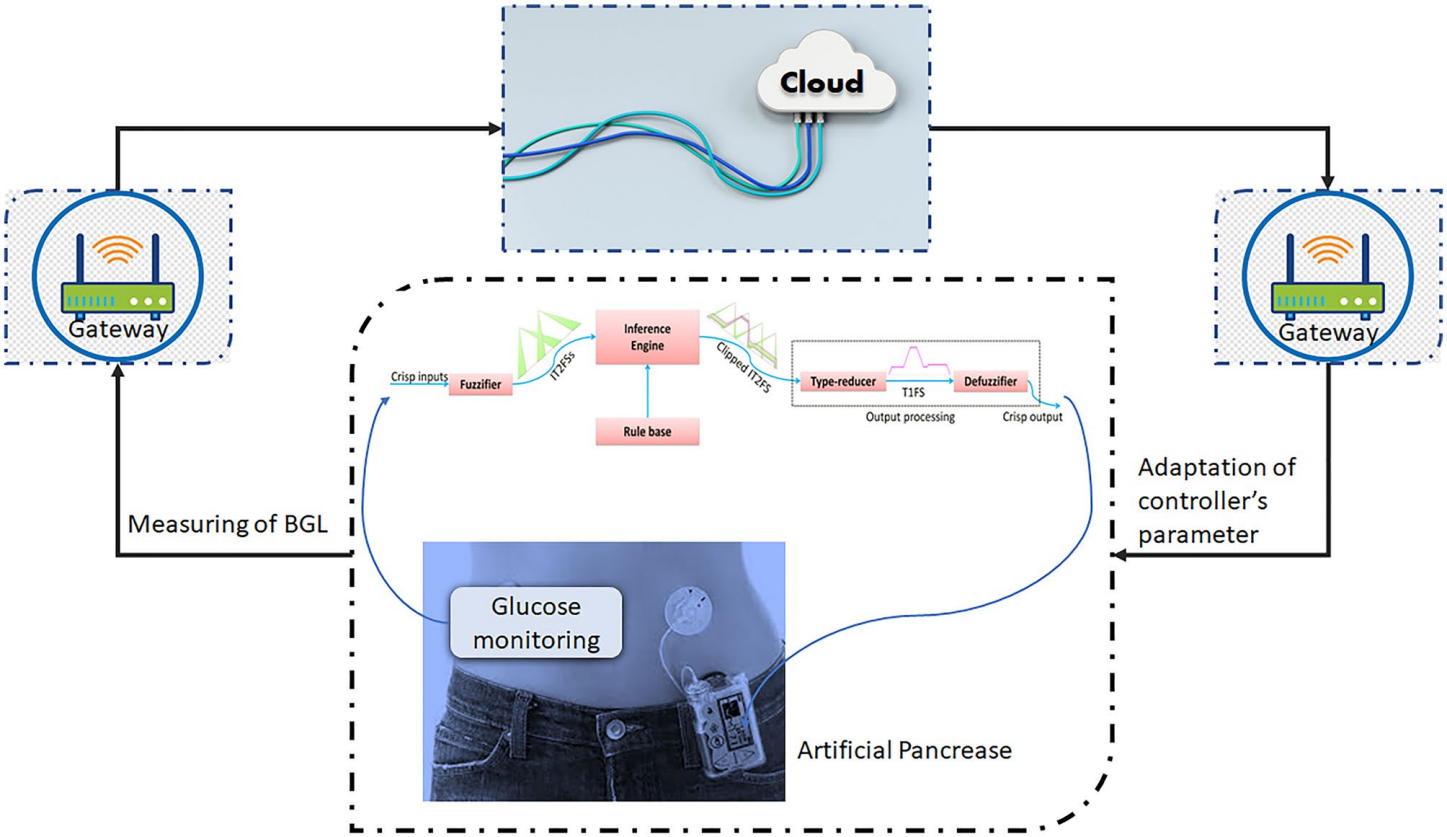
System overview of proposed IoMT.

Artificial Pancreas is a closed loop control systemin which insulin is infused automatically by infusion pump according to both the readings of a continuous glucose sensor (CGS) and the control algorithm that calculates the optimal rate of exogenous insulin infusion^4^. Designing a closed loop AP is important and is primarily depend on the mathematical model of T1DP. But, unfortunately it faces a great challenges when it is applied in practice to a real T1DP because of two reasons: the first reason from the patient side like parametric uncertainty (the parameters of the model vary from patient to patient as well as the parameters vary within the same patient), uncertain meal disturbance, and physical exercise that can burn glucose, thus the dose of insulin needs to be continually changed^6^. And the second reason is related to uncertainties that affect the whole control system in general. So the AP can link to a network of medical devices and share real-time data, shown in Fig. 1, with healthcare professionals thanks to the integration of the Internet of Medical Things (IoMT).

Internet of Medical Things nowadays is a fascinating and quickly developing area in the healthcare industry due to rapid innovation in Information and communication technology^7^. It has the potential to revolutionize healthcare delivery and enhance patient outcomes. IoMT is a network of interconnected sensors, software programs, and medical devices that can talk to one another and share information via the internet to remotely monitor and manage patients' medical problems, deliver more individualized treatment, and cut costs. With the use of IoMT, medical professionals can monitor a patient's vitals, medication compliance, and other health-related data in real-time, enabling early diagnosis of health issues and more proactive treatment. IoMT devices can enable patients to have a more active role in their own health management by giving them access to their health data and enabling them to make educated decisions about their care. So, the IoMT makes it possible for the device to collect a sizable amount of data that can be examined to optimize the controller that give diabetes patients appropriate amount of insulin. Overall, the IoMT technology integration into the AP has the potential to significantly enhance diabetes management and ultimately enhance the quality of life for those who have this chronic condition.

## Related works

Recently, there have been numerous mathematical models that contribute to describe the behavior of glucose-insulin physiological system for T1DP^8,9^, which is in general classified into two types, intravenous, and subcutaneous based on the way of the measurements of insulin and glucose^10^. Many efforts have been made nowadays to design an effective and robust control algorithm for AP based on linear control approaches and nonlinear control approaches. The linear control approach such as PID controllers are given in^2^, that are suggested to prevent excessive intake of exogenous insulin. An adaptive PID algorithm using metaheuristic technique to stabilize the glucose level in blood for T1DP is proposed in^11^. Other controller technique like an inverse optimal control (IOC) that integrates neural identification, a multi-step prediction (MSP) technique, and Takagi–Sugeno (T–S) fuzzy inference is designed in^12^. While the nonlinear control approaches were proposed to regulate the BGL such as terminal synergetic controller and state feedback linearization based controller^13^, arbitrary-order sliding mode based robust controller^14^, backstepping sliding mode controller and super-twisting controller^15^, observer based nonlinear controller^16^, robust observer based adaptive controller^1^, finite‐time robust feedback controller based using backstepping approach^17^, integral backstepping controller, backstepping controller^18^, fuzzy logic based controller^19^.

In most of the aforementioned studies, an intravenous glucose tolerance test (IVGTT) of Bergman minimal model^9^ is considered the most abundant in research related to artificial pancreas, as it is less complex compared to the others while maintaining the minimum state variables necessary to constitute the complete behavior of glucose-insulin physiological system for T1DP. It consists of three differential equations with three states; the first refers to BGL, the second refers to remote insulin, and the latter refers to plasma insulin level. The controllers were created using a Bergman minima model in the majority of the research described above. The mathematical model of glucose-insulin in diverse situations, however, might not be accurate. Also, many conventional methods do not guarantee stability and amny research use evolutionary-based controller learning techniques, which have a high computing cost and lack any stability requirements. Besides, large dynamic disturbances are ignored in most controller techniques.

When the dynamics of the system to be controlled are too complex, nonlinear, time varying, and the system has a large number of inputs and outputs and it has a time delay^20,21^, many variables will affect the performance of the system. Thereby the derivations of the mathematical model of the system which is may deduced from (mechanical laws, physical laws, and so on) in the form of differential and difference equations become too complicated and challenging, and in most situations it would be imprecise to constitute the complete behavior of the real system.

Based on the foregoing, linear control approaches which in many cases depend primarily on the mathematical model of the system would fail to meet the desired performance of the controlled system^22–26^.So, type-2 fuzzy logic controller (T2FLC) which is a type-2 Fuzzy based controller^27,28^ is very appropriate to obtain the desired performance of the controlled system due to the internal structure of type-2 fuzzy logic system (T2FLS) which have the capability to model the uncertainties in the system and minimize the effect of it^29–32^.

Uncertainties are often found in the control system in more than one aspect, we will list some of them: (1) the lack of precision and accuracy of the sensors which are used to support the system with the needed measurements, thereby the data based on these measurements are noisy. (2) Disturbances caused by the surrounding environment such as electromagnetic interference which is related to the stability of the system since it affects directly on data transmission from sensors to controller and from controller to actuators. (3) In fuzzy logic system (FLS) based controller, we use linguistic variables in the antecedent and consequent part of rule base which means that we express variables with words rather than number, these words can be a rich source of uncertainties as words can mean different things to different people (4) Modeling of complex-nonlinear systems causes modeling uncertainties^20,21,33^.

In this study an interval type-2 fuzzy based controller is proposed with the conjunction of meta-heuristic method called grey wolf optimized^34^ for regulation of BGL for T1DP. The extended Bergman minimal model (EBMM) with uncertainty is used to represent the blood glucose dynamic behavior of T1DP^16^. The superiority of IT2FLC is minimizing the effect of uncertainties in the system which is depending primarily on the selecting of footprint of uncertainty (FOU) of interval type-2 fuzzy set (IT2FS) for each inputs and output of the proposed controller. So, these FOUs of IT2FLC are tuned using the GWO and the objective function of GWO is minimized during the optimization process in order to decrease the effect of both parametric uncertainty and uncertain meal disturbance to stabilize BGL within the standard safe level. Thereby, GWO contributes essentially in regulation of BGL for T1DP. Then, the performance and the effectiveness of the proposed controller are studied under four different cases of T1DP using Matlab/Simulink platform. Morever control variability grid analysis (CVGA) is used to evaluate the quality of the proposed controller within 24 h of 100 typ1 1 patients. Three distinct meals 5 mg/dl/min for breakfast, 8 mg/dl/min for lunch, and 8 mg/dl/min dinner are provided for each of 100 T1DPs at 480 min (8 am), 720 min (12 am), and 1200 min (8 am) respectively to observe the response and the efficiency of the optimized IT2FLC under different conditions for various patients. Simulation results show that the proposed controller can avoid both severe hypoglycemia and hyperglycemia for nominal parameters of the model, in addition to model under the presence of both parametric uncertainty and uncertain meal disturbance. The contributions of this work are:

- An effective IT2F controller for Extended Bergman minimal model of T1DP is developed
- An optimal fuzzy compensator is created while taking into account the difficult conditions, including estimating errors, meal impact, and dynamic disturbance by noisy signals.
- The FOU for IT2F controller is tuned using GWO, that can perfectly manage the uncertainty and perturbations in the model
- Conducting several scenarios for different patients to show the superior and performance of the proposed controller

The rest of this paper is organized as follows: in Section “Methods”, we introduce the EBMM of T1DP, the related concepts of IT2FS and metaheuristic technique called GWO is introduced. The simulation of different scenarios for blood glucose situation based on IT2 FSs is presented in Section “Proposed system for regulation of BGL”; Section “Simulation results” provides the four different scenarios for Type-1 diabetic patients; Section “Discussion” provides discussion for the simulation results and analyzes the evolution process of blood glucose situation; Section “Conclusions” concludes the paper.

## Methods

### Extended Bergman minimal model of T1DP

The original mathematical model of Bergman minimal model is consists of three differential equations with three state variables, the first refers to BGL^9^, the second refers to remote insulin, and the latter refers to plasma insulin level, Bergman minimal model has been extended to include fourth equation with fourth state variable to represent the dynamics of meal disturbance, thereby the state space representation of the nonlinear EBMM is as follow^16^:

1 $$\dot{{x}_{1}}=-{p}_{1}\left({x}_{1}-{G}_{b}\right)-{x}_{1}{x}_{2}+{x}_{4}$$

2 $$\dot{{x}_{2}}=-{p}_{2}{x}_{2}+{p}_{3}({x}_{3}-{I}_{b})$$

3 $$\dot{{x}_{3}}=-{p}_{4}\left({x}_{3}-{I}_{b}\right)+u(t)$$

4 $$\dot{{x}_{4}}=-{p}_{5}{x}_{4}$$

where, ${x}_{1}$ refers to the BGL (mg/dl), ${x}_{2}$ refers to the remote insulin (${\mathrm{min}}^{-1}$), ${x}_{3}$ refers to the plasma insulin level (mU/l), ${x}_{4}$ refers to the meal disturbance (mg/dl/min), the initial state of ${x}_{4}$ refers to amount of glucose in the meal, $u(t)$ refers to the rate of exogenous insulin infusion which replaces the natural insulin in the normal body $(\mathrm{mU}/\mathrm{l}/\mathrm{min})$, ${G}_{b}$ refers to the basal value of BGL which expresses the value at which the BGL stabilizes after glucose is absorbed, ${I}_{b}$ refers to the basal value of plasma insulin level which expresses the value at which insulin level stabilizes after insulin is released to lower BGL in blood stream ,${p}_{1}$ refers to glucose effectiveness $({\mathrm{min}}^{-1})$, the ratio $\frac{{p}_{3}}{{p}_{2}}$ refers to insulin sensitivity $(\frac{l}{(mU )})$, ${p}_{4}$ refers to insulin degradation rate $({\mathrm{min}}^{-1})$, and ${p}_{5}$ refers to the rate of appearance of meal disturbance in plasma glucose ${(\mathrm{min}}^{-1})$.

The nominal values and range of parameters for the extended Bergman minimal model (EBMM) are shown in Table 1^16^.

**Table 1:** Nominal value and range for parameters of extended Bergman minimal model (EBMM).

Parameters	Nominal value	Range
${p}_{1}$	$1*{10}^{-7}$	$[0.8*{10}^{-7},1.2*{10}^{-7}]$
${p}_{2}$	0.015	$[0.0105, 0.0195]$
${p}_{3}$	$2*{10}^{-6}$	$[1.4*{10}^{-6}, 2.6*{10}^{-6}]$
${p}_{4}$	0.2	[0.14, 0.26]
${p}_{5}$	0.05	[0.04, 0.06]

### Design of interval T2FLC for regulation of BGL of T1DP

In 1975 Zadeh realized the deficiencies of type-1fuzzy sets (ordinary fuzzy sets) in handling uncertainties in the system. After that the concept of T2FSs is introduced as an extension of the concept of ordinary fuzzy sets^27^. It is well known that a FLS is characterized by its fuzzy sets, so a FLS that is described using at least one T2FS in antecedent or consequent part of its rule-base is called T2FLS^28^. The feasibility of T2FLS appears strongly in the applications that subjected to uncertainties, so many efforts have been made to get benefits from T2FLS and to facilitate the complex mathematical operations associated with it so that researchers and engineers can use it in solving problems of many different applications in various areas.

#### Type-2 fuzzy sets

**Figure 2:**
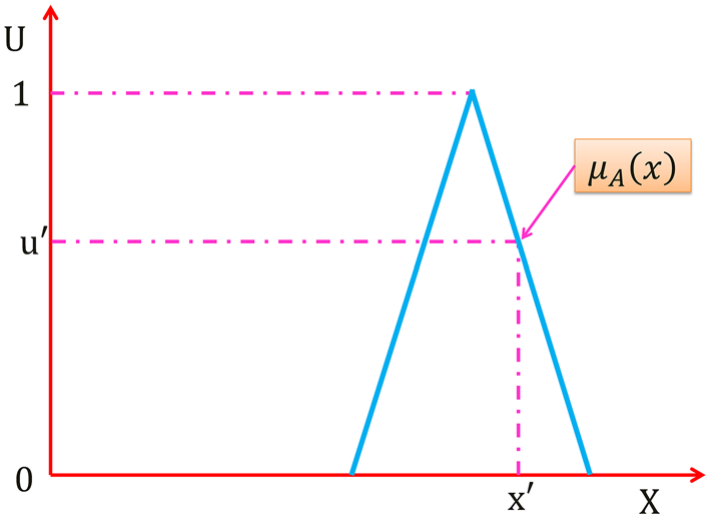
Type-1 fuzzy set.

Consider the triangle T1FS $$A$$ in Fig. 2, the membership function of any given point on the universe of discourse ($$X)$$ $$x{\prime}\in X$$ is a single specific number $${u}{\prime}$$ where $${\mu }_{A}\left({x}{\prime}\right)={u}{\prime}$$. And as explained in details in^28^, So in order to model uncertainties about any variable, It is required a way to measure the dispersion about the variable (how the points that constitute the uncertainties about the variable are distributed and spanned), as in probability density function, variance is needed to provide a measure of dispersion about the mean, so a set of numbers is needed rather than a single specific number in T1FS to capture more information about uncertainties, consequently. T1FSs has limited capabilities to model such uncertainties.

**Figure 3:**
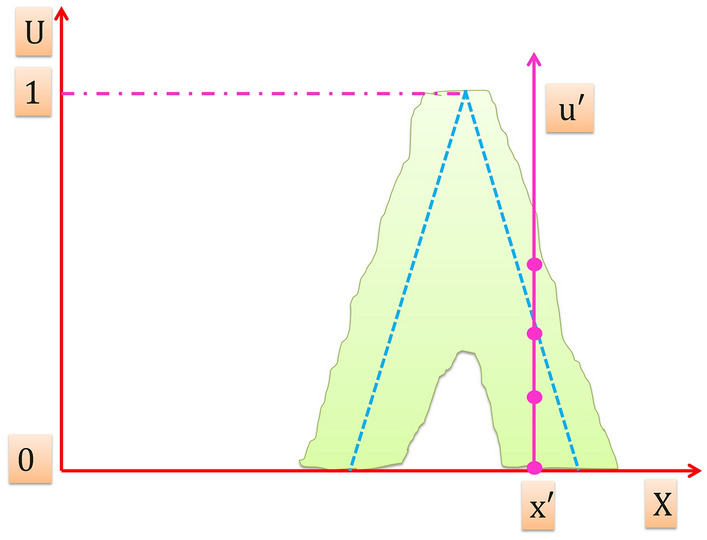
Blurred type-1 membership function.

On the other hand, T2FS have the capability to effectively model the uncertainties in the system, T2FS is obtained with blurring the type-1 membership function of T1FS in Fig. 2 by shifting the points on the triangle either to the left or to the right and not necessarily by the same amounts as illustrated in Fig. 3. Then, at a specific value of $X$ say $x{\prime}\in X$, there is no longer is a single value for the membership function $({u}{\prime})$; instead, the membership function takes on values wherever the vertical line intersects the blur. Those values don’t need be weighted in the same way; hence, it can be assigned an amplitude distribution to all of those points. Doing this for all $x\in X$, a three-dimensional membership function—a type-2 membership function – is created that characterizes a T2FS.”

**Figure 4:**
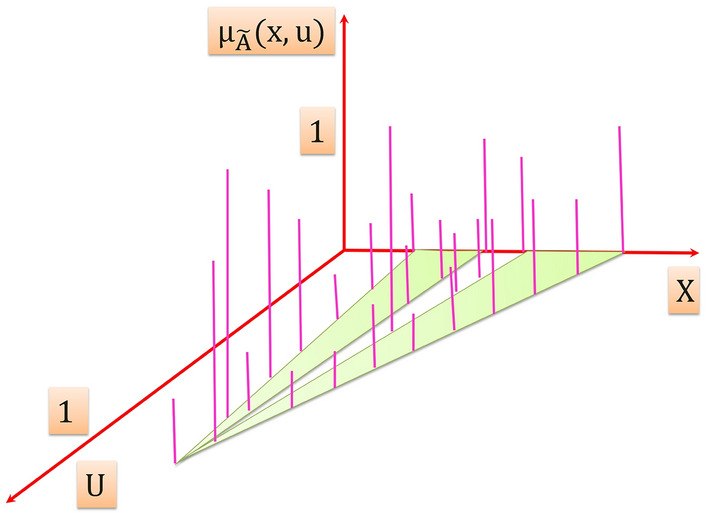
Three dimension type-2 membership function.

Consequently it is obtained three dimensional type-2 membership function its axes are $x, u, {\mu }_{\widetilde{A}}(x,u)$ as illustrated in Fig. 4. Therefore, due to the new third dimension, T2FS can capture more information about the uncertainties about the variable, and thus it can effectively model uncertainties in the system.

**Figure 5:**
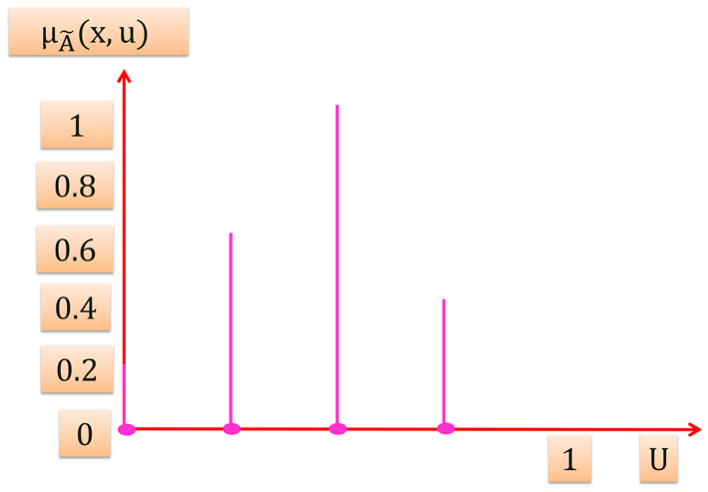
The amplitudes distribution.

Figure 5 illustrates in ${\mu }_{\widetilde{A}}(x,u)-u$ plane that the amplitudes distribution resulted from the intersection of the vertical line at ${x}{\prime}$ with the blurred membership function in Fig. 3 is not necessary that all of them be equal. However when all amplitudes distribution (vertical slices) in ${\mu }_{\widetilde{A}}(x,u)$ axis equal to 1, then the resulting type-2 membership function is an interval type-2 membership function (T2MF) that characterizes an interval T2FS.

**Figure 6:**
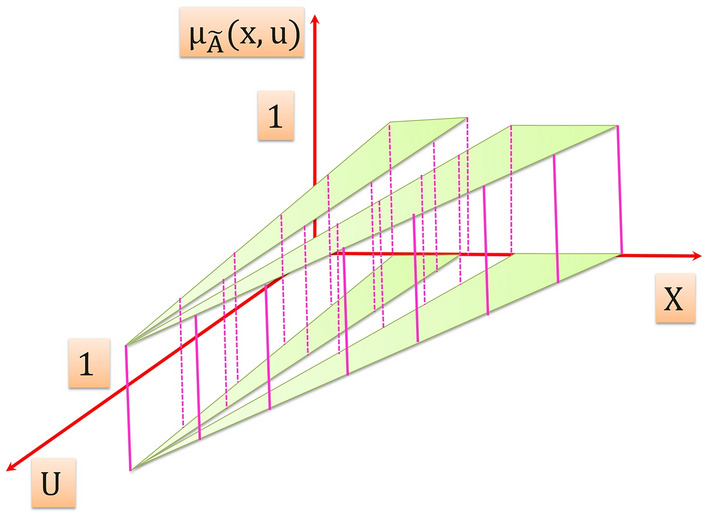
The interval type-2 membership function.

Figure 6 illustrates the three dimensional IT2MF whose axes are $x, u, {\mu }_{\widetilde{A}}(x,u)$ in which all amplitudes distribution (the vertical slices) in ${\mu }_{\widetilde{A}}(x,u)$ axis equal to 1.

Here is the following some notation and definitions listed with comprehensive details in^28^, we will mention some briefly, it will help us to use and communicate about T2FSs:

In Fig. 2 if A is a T1FS, it may be expressed as:

5 $$A=\left\{\left(x,{\mu }_{A}\left(x\right)\right) \forall x \epsilon X\right\}$$

And also can be expressed as:

6 $$A = \int\limits_{x \in X} {\frac{{ \mu_{A} \left( x \right)}}{x}}$$

where ${\mu }_{A}(x)$ is the membership grade of $x\in X$ in $A$ which is a single specific number, it takes just one value from the constraint $0\le {\mu }_{A}(x)\le 1$, and $\int$ denotes union over all admissible *x*.

In Fig. 4$\widetilde{A}$ is a T2FS, is expressed by:

7 $$\widetilde{A}=\left\{\left(\left(x,u\right),{\mu }_{\widetilde{A}}\left(x,u\right)\right) \forall x \epsilon X , \forall u \epsilon { J}_{x} \subseteq \left[0, 1\right]\right\}$$

In which $$0\le {\mu }_{\widetilde{A}}(x,u)\le 1$$

Also $\widetilde{A}$ can be expressed as:

8 $$\tilde{A} = \int\limits_{x \in X} {\int\limits_{u \in U} {\frac{{\mu_{{\tilde{A}}} \left( {x,u} \right)}}{{\left( {x,u} \right)}}\;J_{x} \subseteq \;\left[ {0,1} \right]} }$$

where $\mu_{{\tilde{A}}} \left( {x,u} \right)$ is a type-2 membership function that characterizes a T2FS, and $\iint$ denotes union over all admissible $x$ and $u$.

${J}_{x}$ Is the primary membership of $x$ in $\widetilde{A}$, also called the domain of a secondary membership of $x$ in $\widetilde{A}$.

The memberships of the primary membership of $x$ in $\widetilde{A}$ are called the secondary membership of $x$ in $\widetilde{A}$ and also called the vertical slices in ${\mu }_{\widetilde{A}}\left(x,u\right)$ axis.

The amplitude of a secondary membership is called a secondary grade.

When all of the vertical slices in ${\mu }_{\widetilde{A}}\left(x,u\right)$ axis equal 1 (the secondary grades equal 1), then the resulting T2MF is an interval T2MF that characterizes an interval T2FS, $\tilde{A}$ can be expressed by:

9 $$\tilde{A} = \int\limits_{x \in X} {\int\limits_{u \in U} {\frac{1}{{\left( {x,u} \right)}}\;J_{x} \subseteq \;\left[ {0,1} \right]} }$$

The FOU in the primary memberships of $\widetilde{A}$ is the union of all primary memberships that is:

10 $$FOU\left( {\tilde{A}} \right) = \bigcup\nolimits_{x \in X} {J_{x} }$$

**Figure 7:**
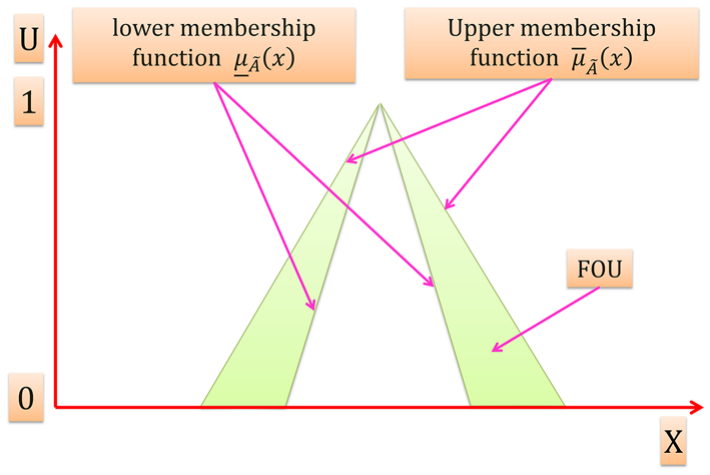
FOU for an interval T2FS.

The shaded region on the $x-u$ plane in Fig. 4 is the FOU. For an interval T2FS the FOU is a complete description of an interval T2FS, this is because the secondary grades of an interval T2FS equal to 1, thereby it conveys no new information. The FOU for an interval T2FS is shown in Fig. 7, it is bounded from the above and below by a two type-1 membership functions that are called lower membership function and upper membership function, and are denoted $\overline{\mu _{\widetilde{A}}}\left(x\right)$, $\underset{\_}{{\mu }_{\widetilde{A}}}\left(x\right)$, $\forall x \epsilon X$ respectively.

#### Interval type-2 fuzzy logic system

Unfortunately the mathematical operations that are associated with T2FSs are very complex and time consuming while it is simple to interval T2FSs^28^ which is considered a special case of the general T2FSs as it needs to can use interval T2FSs that we are just familiar with the mathematical operations of T1FSs such as: union, intersection, and complement. Consequently interval T2FLS that is characterized by its interval T2FS have gained popularity among many practitioners and engineers, and for this, interval T2FLS have been contributed in different applications in different fields^32^.

**Figure 8:**
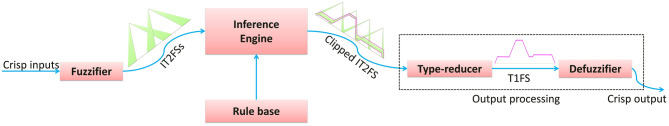
the structure of interval T2FLS.

The structure of interval T2FLS is shown in Fig. 8 ,it is almost the same as for its counterpart T1FLS, its rule-base is composed of (IF–THEN) rules as for T1FLS but now some or all of its antecedent and consequent parts are of interval T2FS, it differs from T1FLS in that the output of the fuzzifier block is now of interval T2FSs, consequently the fuzzy output sets of inference engine block is now of interval T2FSs, and as the case for most applications like in a control system, it requires a single crisp number from the output of FLS rather than a set of numbers in order to can take a control action, so the defuzzifier block in T1FLS is replaced by output processing block which internally consists of two blocks, the first which converts interval T2FS to T1FS is called type-reducer and the second is just a defuzzifier as in T1FLS to convert T1FS to a single crisp number.

In general there are two ways to go from interval T2FSs to a crisp number, the first one is to use type-reducer + defuzzification method which first converts interval T2FSs to T1FS then a defuzzification is run to convert T1FS to a crisp number, the second is called direct defuzzification method which converts interval T2FSs directly to a crisp number without the need of using a type-reduction, in this paper we will use type-reducer + defuzzification method.

There are many algorithms one can use to perform type-reduction, Karnik–Mendel (KM) algorithm^28^ is the popular one, it is simple and converges to the exact solution, but it is iterative and computationally intensive, so in the last few years a lot of researches have been provided in attempt to reduce the computational cost associated with KM algorithm such as enhanced KM algorithm which can reduce more than 39% in computational time over KM algorithm, and an enhanced iterative algorithm with stop condition (EIASC) which can save more 50% computational cost over KM algorithm particularly when number of rules $N\le 100$.

Also for Mamdani interval T2FLS, Wu- Mendel proposed a method called the Wu-Mendel uncertainty bounds to approximate the type -reduced set to avoid the iterative algorithms, it calculates four centroids which is called (boundary type-1 fuzzy systems) for an interval T2FLS, it depends only on the upper and lower firing level of each rule and the endpoints centroids of each rule consequent, but in this method we still have to use KM algorithm to obtain the endpoints centroids of each rule consequent which have to be done once.

In this paper we will focus on using EIASC algorithm to perform type-reduction to get a crisp number from interval T2FLS.

Now in the following subsections we will explain in details how to implement each stage of interval T2FLS that will later incorporate into interval T2FLC for regulation of BGL of T1DP to get a crisp number from intervalT2FSs, and for further clarification we will support the explanation of each following stage with a specific numerical example that will last during the explanation of each stage.

#### Rule-base of interval TFLS

In this paper we will use Zadeh rule-base, and as we mentioned earlier the structure of rule-base for interval T2FLS remains the same as for T1FLS counterpart, the only difference is some or all of its antecedent and consequent parts are of interval T2FS, the structure of the general Zadeh rule for interval T2FLS which has $p$ inputs ${x}_{1}\in {X}_{1},\dots .,{x}_{p}\in {X}_{p}$, and one output $y\in Y$ is as follow^28^:

$$R^{l} :IF x_{1} is \tilde{F}_{1}^{l} and \ldots and x_{p } is \tilde{F}_{p}^{l} , THEN y is \tilde{G}^{l} ,\quad l = 1, \ldots , N.$$

where ${\varvec{N}}$ is number of rules, ${\varvec{l}}$ is the rank of rule, ${\widetilde{{\varvec{F}}}}_{1}^{{\varvec{l}}}\dots \dots ..{\widetilde{{\varvec{F}}}}_{{\varvec{p}}}^{{\varvec{l}}}$ are interval T2FSs, and ${\widetilde{{\varvec{G}}}}^{{\varvec{l}}}$ is interval T2FS which is an interval set is expressed in the form of endpoints centroid of a consequent of interval T2FS, where ${\widetilde{{\varvec{G}}}}^{{\varvec{l}}}=[{\underset{\_}{{\varvec{G}}}}^{{\varvec{l}}},{\overline{{\varvec{G}}^{\varvec{l}}}}]$.

**Figure 9:**
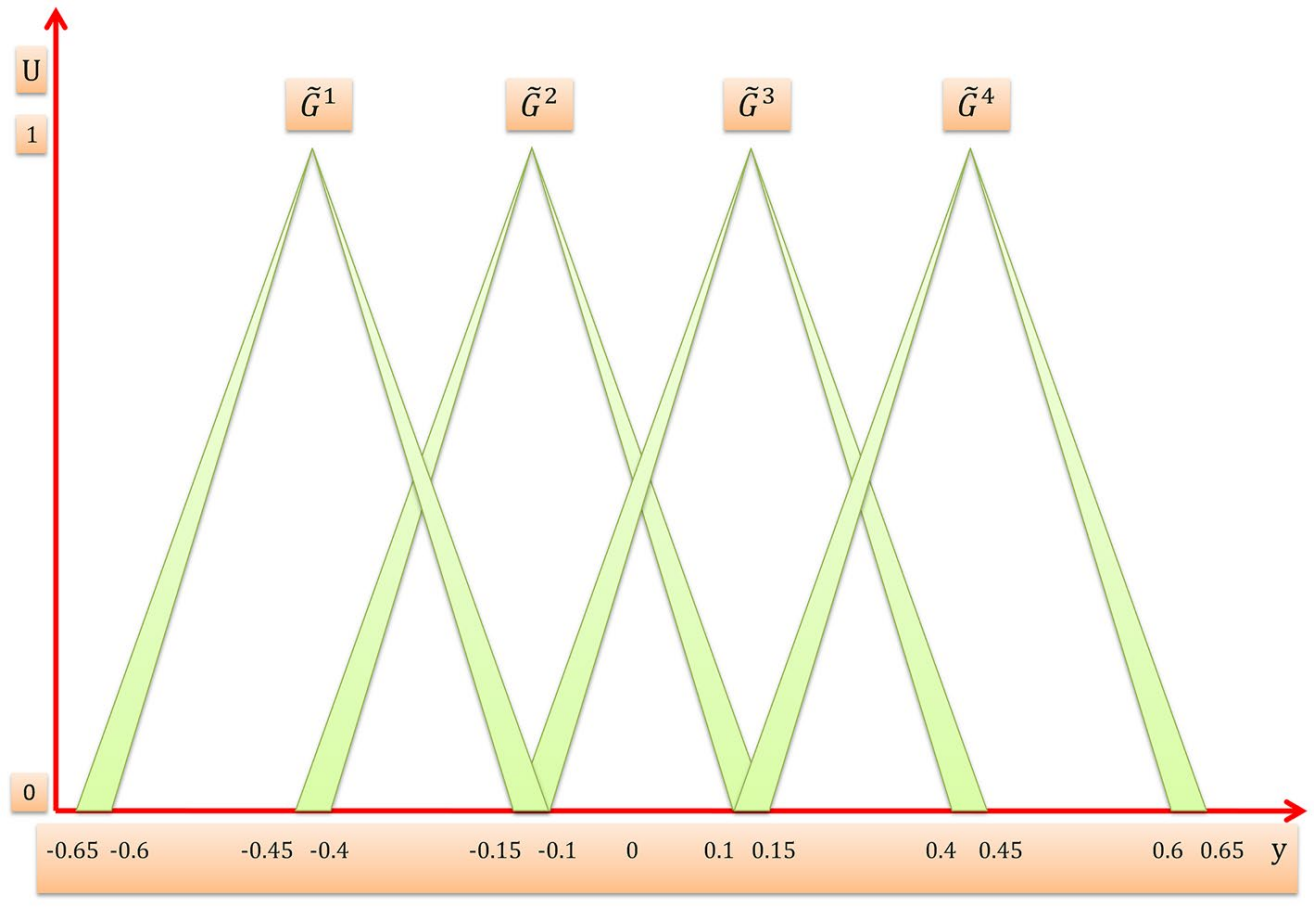
Interval T2MFs of output ${\varvec{y}}$.

For example we suppose that we have an interval T2FLS with four rules $N=4$, two inputs ${x}_{1}$,${x}_{2}$ and one output $y$, the domain of input ${x}_{1}$ consists of two triangular interval T2MFs $\widetilde{A}$ and $\widetilde{B}$, the domain of input ${x}_{2}$ consists of two triangular interval T2MFs $\widetilde{C}$ and $\widetilde{D}$, and the domain of output $y$ consists of four triangular interval T2MFs ${\widetilde{G}}^{1}$,${\widetilde{G}}^{2}$, ${\widetilde{G}}^{3}$, and ${\widetilde{G}}^{4}$ as shown in Fig. 9, then the four rules are:

$$\begin{aligned} & R^{1} :IF x_{1} is \tilde{A} and x_{2} is \tilde{C}, THEN y is \tilde{G}^{1} \\ & R^{2} :IF x_{1} is \tilde{A} and x_{2} is \tilde{D}, THEN y is \tilde{G}^{2} \\ & R^{3} :IF x_{1} is \tilde{B} and x_{2} is \tilde{C}, THEN y is \tilde{G}^{3} \\ & R^{4} :IF x_{1} is \tilde{B} and x_{2} is \tilde{D}, THEN y is \tilde{G}^{4} \\ \end{aligned}$$

#### Fuzzifier of interval T2FLS

It performs fuzzification process which converts crisp inputs into interval T2FSs, it is the same as for the fuzzification of T1FSs, but now we have for each point in the universe of discourse two interval membership values rather than one membership value in the T1FSs, both of them are calculated from the intersection of the vertical line with the lower membership function and upper membership function respectively. For example when ${x}_{1}={x}_{1}{\prime}=-0.07$ and ${x}_{2}={x}_{2}{\prime}=0.09$ , then the vertical line at ${x}_{1}{\prime}$ intersects $\widetilde{A}$ within the two interval values $\left[{\underset{\_}{\mu }}_{\widetilde{A}} \left({x}_{1}{\prime}\right),{\overline{\mu_{\widetilde{A}}}} \left({x}_{1}{\prime}\right)\right]$ also intersects $\widetilde{B}$ within the two interval values $\left[{\underset{\_}{\mu }}_{\widetilde{B}} \left({x}_{1}{\prime}\right),{\overline{\mu_{\widetilde{B}}}} \left({x}_{1}{\prime}\right)\right]$, the vertical line at ${x}_{2}{\prime}$ intersects $\widetilde{C}$ within the two interval values $\left[{\underset{\_}{\mu }}_{\widetilde{C}} \left({x}_{2}{\prime}\right),{\overline{\mu_{\widetilde{C}}}} \left({x}_{2}{\prime}\right)\right]$ also intersects $\widetilde{D}$ within the two interval values $\left[{\underset{\_}{\mu }}_{\widetilde{D}} \left({x}_{2}{\prime}\right),{\overline{\mu_{\widetilde{D}}}} \left({x}_{2}{\prime}\right)\right]$, thereby the interval values that deduced from the fuzzification process of the inputs are:

$$\begin{aligned} \mu_{{\tilde{A}}} \left( {x_{1}{\prime} } \right) & = \left[ {\underline {\mu }_{{\tilde{A}}} \left( {x_{1}{\prime} } \right),\overline {\mu }_{{\tilde{A}}} \left( {x_{1}{\prime} } \right)} \right] \\ \mu_{{\tilde{B}}} \left( {x_{1}{\prime} } \right) & = \left[ {\underline {\mu }_{{\tilde{B}}} \left( {x_{1}{\prime} } \right),\overline {\mu }_{{\tilde{B}}} \left( {x_{1}{\prime} } \right)} \right] \\ \mu_{{\tilde{C}}} \left( {x_{2}{\prime} } \right) & = \left[ {\underline {\mu }_{{\tilde{C}}} \left( {x_{2}{\prime} } \right),\overline {\mu }_{{\tilde{C}}} \left( {x_{2}{\prime} } \right)} \right] \\ \mu_{{\tilde{D}}} \left( {x_{2}{\prime} } \right) & = \left[ {\underline {\mu }_{{\tilde{D}}} \left( {x_{2}{\prime} } \right),\overline {\mu }_{{\tilde{D}}} \left( {x_{2}{\prime} } \right)} \right] \\ \end{aligned}$$

#### Inference engine of interval T2FLS

It is the same as for the inference engine of T1FLS, it deduces the clipped interval T2FS consequent for the output variable of each rule in the rule-base for a given crisp value of the input variables (those values that we get from the fuzzifier of interval TFLS stage) and this process also called rule firing, consequently we have for each rule of interval T2FLS two interval firing level, lower firing level ${\underline{f}}^{l}$ and upper firing level ${\overline{f}}^{l}$^28^, then it combines all the clipped interval T2FSs consequents of the fired rules into one overall fuzzy set that constitutes the output of the inference engine which is the input to the output processing, We assumed to use interval type-2 mamdani fuzzy system and minimum t-norm operator, so the two firing interval for the four rules are:

$$\begin{aligned} \left[ {\underline {f}^{1} ,\overline{ f}^{1} } \right] & = \left[ min {\left[ {\underline {\mu }_{{\tilde{A}}} \left( {x_{1}{\prime} } \right), \underline {\mu }_{{\tilde{C}}} \left( {x_{2}{\prime} } \right)} \right], min \left[ {\overline {\mu }_{{\tilde{A}}} \left( {x_{1}{\prime} } \right), \overline {\mu }_{{\tilde{C}}} \left( {x_{2}{\prime} } \right)} \right]} \right] \\ \left[ {\underline {f}^{2} ,\overline{ f}^{2} } \right] & = \left[ min {\left[ { \underline {\mu }_{{\tilde{A}}} \left( {x_{1}{\prime} } \right), \underline {\mu }_{{\tilde{D}}} \left( {x_{2}{\prime} } \right)} \right],min \left[  { \overline {\mu }_{{\tilde{A}}} \left( {x_{1}{\prime} } \right), \overline {\mu }_{{\tilde{D}}} \left( {x_{2}{\prime} } \right)} \right]} \right] \\ \left[ {\underline {f}^{3} ,\overline{ f}^{3} } \right] & = \left[ min {\left[ {\underline {\mu }_{{\tilde{B}}} \left( {x_{1}{\prime} } \right),\underline {\mu }_{{\tilde{C}}} \left( {x_{2}{\prime} } \right)} \right], min \left[ { \overline {\mu }_{{\tilde{B}}} \left( {x_{1}{\prime} } \right),\overline {\mu }_{{\tilde{C}}} \left( {x_{2}{\prime} } \right)} \right]} \right] \\ \left[ {\underline {f}^{4} ,\overline{ f}^{4} } \right] & = \left[ min {\left[ {\underline {\mu }_{{\tilde{B}}} \left( {x_{1}{\prime} } \right) \cdot \underline {\mu }_{{\tilde{D}}} \left( {x_{2}{\prime} } \right)} \right],min \left[  {\overline {\mu }_{{\tilde{B}}} \left( {x_{1}{\prime} } \right),\overline {\mu }_{{\tilde{D}}} \left( {x_{2}{\prime} } \right)} \right]} \right] \\ \end{aligned}$$

**Figure 10:**
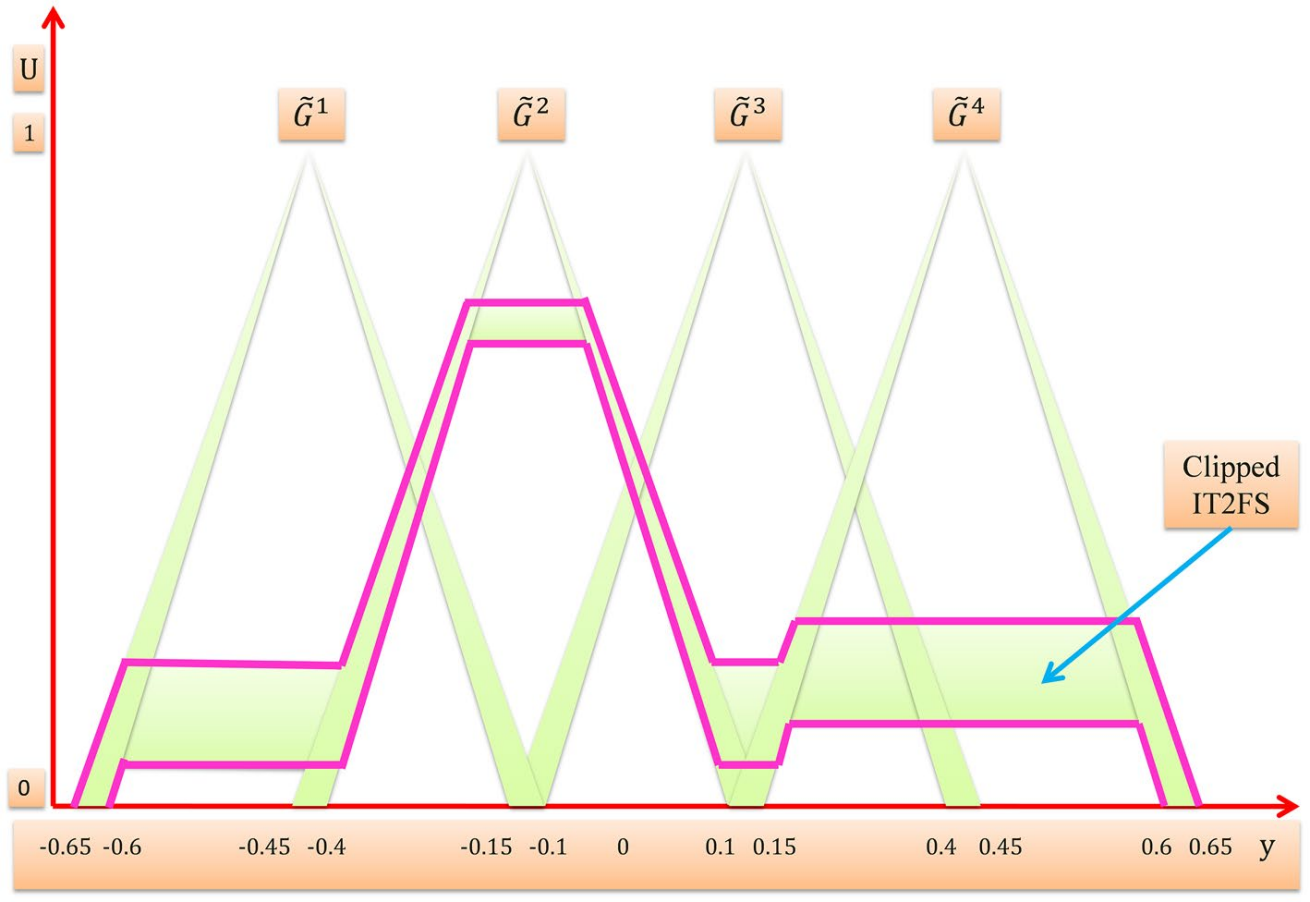
Overall clipped interval T2FS.

Now we have got the values for the firing interval of each rule, then ${\underline{f}}^{l}$ for each rule is t-normed with lower membership function of ${\widetilde{G}}^{l}$ and ${\overline{f}}^{l}$ for each rule is t-normed with upper membership function of ${\widetilde{G}}^{l}$ to get a clipped interval T2FS consequent from each rule, and after that the clipped interval T2FS consequent of each rule are combined together (using union operator) into one overall clipped interval T2FS as shown in Fig. 10.

#### Type-reduction and defuzzification of IT2FSs

It was supposed just as for T1FLS after we got the resulted clipped interval T2FS that we can as usual run one of the defuzzification methods to get the crisp output, but for the interval T2FLS the union of the clipped interval T2FSs of each rule become a big issue with regarding to the computational complexity as it requires additional computational time and memory storage particularly with a real time applications such as a control system^31^.

So instead of type-reducer + defuzzification method is used to combine the firing interval of each rule with the corresponding rule consequent and get the crisp output, there are many methods to do that: Centroid, Height, and Center-of- sets type-reducer + defuzzification method. In this paper we will use center-of-sets (COS) method for an interval type-2 Mamdani fuzzy system in which the firing interval of each rule is combined with the corresponding endpoints centroids of its consequent.

By using this method we get the COS type-reduced-set denoted ${Y}_{cos}\left({x}{\prime}\right)$ for an interval type-2 mamdani fuzzy system Where:

11 $$Y_{\cos } \left( {x^{\prime } } \right) = \frac{1}{{\left[ {y_{l} ,y_{r} } \right]}},$$

12 $$y_{l} = \frac{{\mathop \sum \nolimits_{l = 1}^{L} \overline {f}^{l} \underline {G}^{l} + \mathop \sum \nolimits_{l = L + 1}^{N} \underline {f}^{l} \underline {G}^{l} }}{{\mathop \sum \nolimits_{l = 1}^{L} \overline {f}^{l} + \mathop \sum \nolimits_{l = L + 1}^{N} \underline {f}^{l} }},$$

13 $$y_{r} = \frac{{\mathop \sum \nolimits_{l = 1}^{R} \underline {f}^{l} \overline {G}^{l} + \mathop \sum \nolimits_{l = R + 1}^{N} \overline {f}^{l} \overline {G}^{l} }}{{\mathop \sum \nolimits_{l = 1}^{R} \underline {f}^{l} + \mathop \sum \nolimits_{l = R + 1}^{N} \overline {f}^{l} }},$$

N is number of rules; L and R are the switch points that

Satisfy:

$$\begin{aligned} \underline {G}^{L} & \le y_{l} \le \underline {G}^{L + 1} \\ \overline {G}^{R} & \le y_{r} \le \overline {G}^{R + 1} \\ \end{aligned}$$

${y}_{l}$ and ${y}_{r}$ can be computed using EIASC algorithm , after that we can easily get the crisp output of interval type-2 mamdani fuzzy system from the following formula

14 $$y=\frac{{y}_{l}+{y}_{r}}{2}.$$

But before we go through EIASC algorithm we have to first calculate for each rule the endpoints centroids of its consequent by using the KM algorithm which is calculated only once using the Matlab function “centroidIT2”^35^ as follow:

$$\begin{aligned} \tilde{{G}}^{1} & = \user2{ }\left[ {{\underline {G} }^{1} ,{ \overline {G} }^{1} } \right] \\ \tilde{G}^{2} & = \left[ {\underline {G}^{2} , \overline {G}^{2} } \right] \\ \tilde{G}^{3} & = \left[ {\underline {G}^{3} , \overline {G}^{3} } \right] \\ \tilde{G}^{4} & = \left[ {\underline {G}^{4} , \overline {G}^{4} } \right] \\ \end{aligned}$$

where ${\underline{G}}^{l},{\overline{G}}^{l}$ are the two endpoints centroids of the consequent ${\widetilde{G}}^{l}$ and $l=1,\dots \dots ,N$.

• EIASC algorithm for computing ${y}_{l}$:

- Sort ${\underline{G}}^{l}$ $(l=1,\dots \dots ,N)$ in increasing order and call the sorted ${\underline{G}}^{l}$ by the same name, but now ${\underline{G}}^{1}\le {\underline{G}}^{2}\le \dots \le {\underline{G}}^{N}$. Match the weights ${F}^{l}$(${x}{\prime}$) with their respective ${\underline{G}}^{l}$ and renumber them so that their index corresponds to the renumbered ${\underline{G}}^{l}$.
- Initialize
  $$\begin{aligned} a & = \mathop \sum \limits_{l = 1}^{N} \underline {G}^{l} \underline {f}^{l} , \\ b & = \mathop \sum \limits_{l = 1}^{N} \underline {f}^{l} , \\ y_{l} & = \underline {G}^{N} ,\quad L = 0. \\ \end{aligned}$$
- Compute
  $$\begin{aligned} L & = L + 1, \\ a & = a + \underline {G}^{L} \left( {\overline {f}^{L} - \underline {f}^{L} } \right), \\ b & = b + \overline {f}^{L} - \underline {f}^{L} , \\ y_{l} & = \frac{a}{b}. \\ \end{aligned}$$
- If ${y}_{l}\le {\underline{G}}^{L+1}$ , stop; otherwise go to step (c).

• EIASC algorithm for computing ${y}_{r}$:

- Sort ${\overline{G}}^{l}$ $(l=1,\dots \dots ,N)$ in increasing order and call the sorted ${\overline{G}}^{l}$ by the same name, but now ${\overline{G}}^{1}\le {\overline{G}}^{2}\le \dots \le {\overline{G}}^{N}$. Match the weights ${F}^{l}$(${x}{\prime}$) with their respective ${\overline{G}}^{l}$ and renumber them so that their index corresponds to the renumbered ${\overline{G}}^{l}$.
- Initialize
  $$\begin{aligned} a & = \mathop \sum \limits_{l = 1}^{N} \overline {G}^{l} \underline {f}^{l} , \\ b & = \mathop \sum \limits_{l = 1}^{N} \underline {f}^{l} , \\ y_{r} & = \overline {G}^{1} ,\quad R = N. \\ \end{aligned}$$
- Compute
  $$\begin{aligned} a & = a + \overline {G}^{R} \left( {\overline {f}^{R} - \underline {f}^{R} } \right), \\ b & = b + \overline {f}^{R} - \underline {f}^{R} , \\ y_{r} & = \frac{a}{b},\quad R = R - 1. \\ \end{aligned}$$
- If ${y}_{r}\ge {\overline{G}}^{R}$, stop; otherwise go to step (c).

Using EIASC algorithm and consequently we can now compute the crisp output of interval type-2 Mamdani fuzzy system from the following formula:

$$y=\frac{{y}_{l}+{y}_{r}}{2}$$

### Grey wolf optimizer

The parameters, $wi, wi1, wi2$ of interval T2FLC are tuned using GWO algorithm^34^. GWO imitates the leadership hierarchy and hunting mechanism of grey wolves in nature, the hunting mechanism includes three steps: (1) Searching and approaching the prey. (2) Encircling, and harassing the prey until it stops moving. (3) Attacking the prey.

The leadership hierarchy of grey wolves in the herd includes four types of grey wolves like alpha, beta, delta, and omega. The dominance of the leadership hierarchy decrease from alpha to omega where alpha is the leader that responsible for making decisions, Beta help alpha in making decisions enhances the commands of alpha in the herd and gives the feedback to alpha, Omega has to submit to alpha, beta and delta, it plays the role of scapegoat, and it is considered the lowest rank in the herd, Delta has to submit to alpha and beta but it dominates omega, it constitutes the category of elders, scouts, caretakers, sentinels, and hunters.

The mathematical model of GWO depends on three main rules:

(1) Searching for prey and Leadership hierarchy

Searching process for prey is always guided by the positions of Alpha ($\alpha$), beta ($\beta$), and delta ($\delta$), grey wolves diverge from each other during searching process and converge to attach prey when it stops moving. So during optimization (hunting) $\alpha$ is considered the best fittest solution, $\beta$ as the second best solution, and $\delta$ as the third best solution, and the rest of solutions is considered as omega ($\omega$) that follows alpha, beta, and delta.

(2) Encircling and harassing prey

Encircling behavior of grey wolves during hunting (optimization) can be modeled with following equations^34^.

15 $$\overrightarrow{D}=\left|\overrightarrow{C}.{\overrightarrow{X}}_{p}(t)-\overrightarrow{X}(t)\right|$$

16 $$\overrightarrow{X}(t+1)=\left|{\overrightarrow{X}}_{p}\left(t\right)-\overrightarrow{A}.\overrightarrow{D}\right|$$

17 $$\overrightarrow{A}=2\overrightarrow{a}.{\overrightarrow{r}}_{1}-\overrightarrow{a}$$

18 $$\overrightarrow{C}=2.{\overrightarrow{r}}_{2}$$

where: $\overrightarrow{A}$,$\overrightarrow{C}$ coefficient vectors,${\overrightarrow{X}}_{p}$ refers to the position vector of the prey, $\overrightarrow{X}$ refers to the position vector of a grey wolf, $t$ refers to the current iteration.

$\overrightarrow{a}$ is decreased linearly from $2$ to $0$ during the optimization, ${\overrightarrow{r}}_{1},{\overrightarrow{r}}_{2}$ are random vectors in [$\mathrm{0,1}$].

(3) Attacking process

Grey wolves update their positions guided by *α*, $\beta$, and $\delta$, they start to attack the prey when it stops moving, the process can be modelled with following equations

19 $${D}_{\alpha }=\left|{\overrightarrow{C}}_{1}.{\overrightarrow{X}}_{\alpha }-\overrightarrow{X}\right|$$

20 $${D}_{\beta }=\left|{\overrightarrow{C}}_{2}.{\overrightarrow{X}}_{\beta }-\overrightarrow{X}\right|$$

21 $${D}_{\delta }=\left|{\overrightarrow{C}}_{3}.{\overrightarrow{X}}_{\delta }-\overrightarrow{X}\right|$$

22 $${\overrightarrow{X}}_{1}={\overrightarrow{X}}_{\alpha }-{\overrightarrow{A}}_{1}.({\overrightarrow{D}}_{\alpha })$$

23 $${\overrightarrow{X}}_{2}={\overrightarrow{X}}_{\beta }-{\overrightarrow{A}}_{2}.({\overrightarrow{D}}_{\beta })$$

24 $${\overrightarrow{X}}_{3}={\overrightarrow{X}}_{\delta }-{\overrightarrow{A}}_{3}.({\overrightarrow{D}}_{\delta })$$

25 $$\overrightarrow{X}\left(t+1\right)=\frac{{\overrightarrow{X}}_{1}+{\overrightarrow{X}}_{2}+{\overrightarrow{X}}_{3}}{3}$$

**Figure 11:**
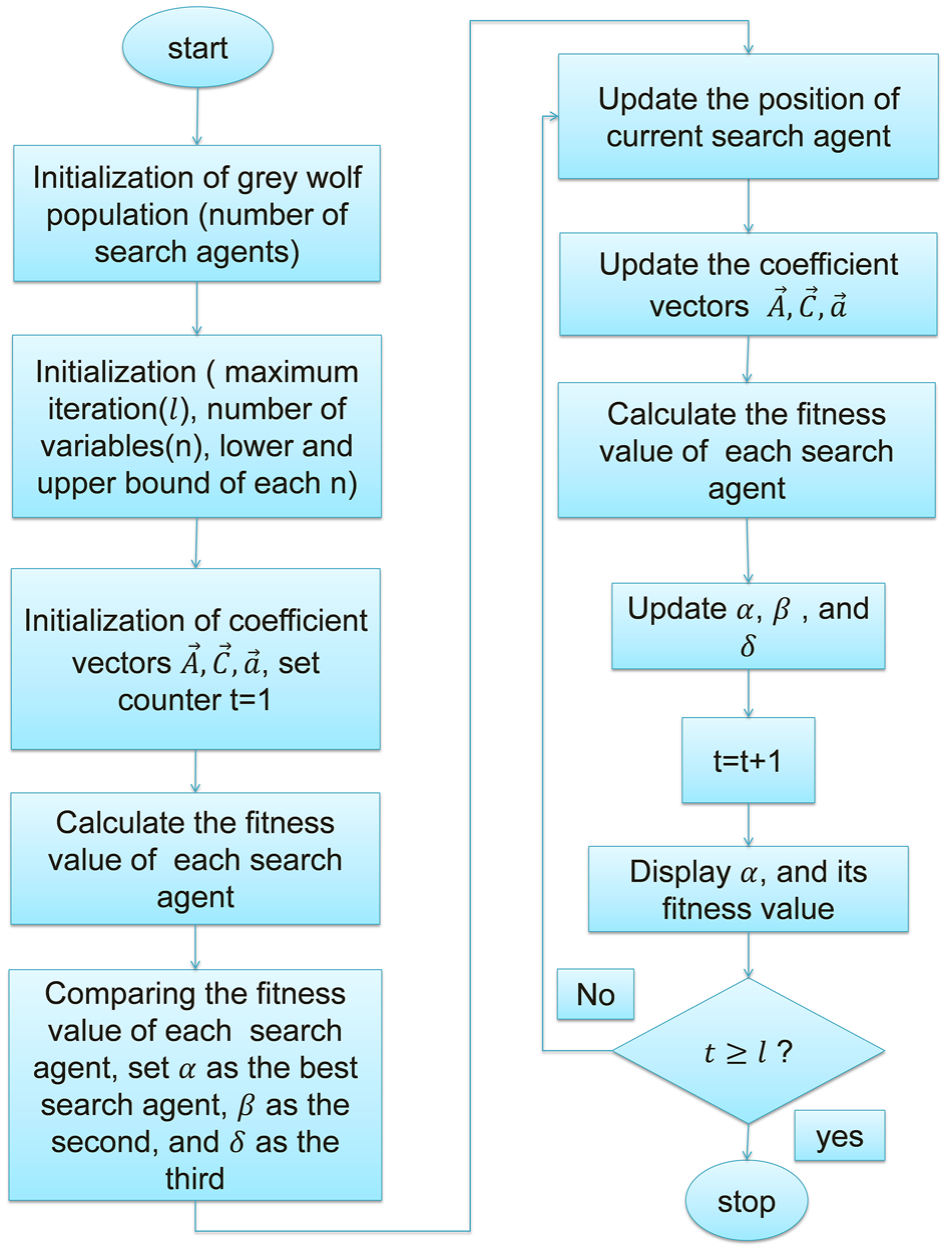
Flow chart of GWO.

The flowchart for the GWO is shown in Fig. 11.

The fitness value for each search agent is calculated from the objective function that is considered the criteria most influential during optimization to obtain the desired parameters of the controller, it may be minimized or maximized to obtain the desired response of the controller.

### Stability analyses

In this section we shall prove asymptotic stability of interval T2FLC for regulation BGL of T1DP using Lyapunov theory^36^. Our target is to design the control signal $U$ such that the BGL of T1DP ${x}_{1}$ follows the set BGL $R$, that is the error signal $e\to 0$ as $t\to \infty$ where:

26 $$e=R-{x}_{1}$$

So the Lyapunov function candidate is chosen as

27 $$V\left(e,\dot{e}\right)=\frac{1}{2}\left({e}^{2}+{\dot{e}}^{2}\right)$$

According to Lyapunov stability theory for the system to be asymptotic stable, $\dot{V}(e,\dot{e})$ must be negative definite^36^.

By taking time derivative of Eq. (27), it yields:

28 $$\dot{V}\left(e,\dot{e}\right)=e\dot{e}+\dot{e}\ddot{e}.$$

By taking the time derivative of Eq. (26), it yields:

29 $$\dot{e}=-\dot{{x}_{1}}$$

By substitution for $\dot{{x}_{1}}$ from Eq. (1) in Eq. (29), it yields:

30 $$\dot{e}={p}_{1}\left({x}_{1}-{G}_{b}\right)+{x}_{1}{x}_{2}-{x}_{4}$$

By taking the second time derivative of Eq. (30), it yields:

31 $$\ddot{e}={p}_{1}\dot{{x}_{1}}+{x}_{1}\dot{{x}_{2}}+\dot{{x}_{1}}{x}_{2}-\dot{{x}_{4}}$$

By substitution for $\dot{{x}_{2}},\dot{{x}_{4}}$ from Eqs. (2), (4) respectively in Eq. (31), it yields:

32 $$\ddot{e}={p}_{1}\dot{{x}_{1}}+{x}_{1}\left(-{p}_{2}{x}_{2}+{p}_{3}{x}_{3}-{p}_{3}{I}_{b}\right)+\dot{{x}_{1}}{x}_{2}+{p}_{5}{x}_{4}$$

By taking the third time derivative of Eq. (32), it yields:

33 $$e^{ \cdots } = p_{1} \ddot{{x}_{1}} - p_{2} x_{1} \dot{{x}_{2}} - p_{2} \dot{{x}_{1}} x_{2} + p_{3} x_{1} \dot{{x}_{3}} + p_{3} \dot{{x}_{1}} x_{3} - p_{3} I_{b} \dot{{x}_{1}} + \dot{{x}_{1}} \dot{{x}_{2}} + \ddot{{x}_{1}} x_{2} + p_{5} \dot{{x}_{4}}$$

By substitution for $\dot{x}_{3}$ from Eq. (3) in Eq. (33), it yields:

34 $$e^{ \cdots } = \ddot{{x}_{1}} \left( {p_{1} + x_{2} } \right) + \dot{{x}_{1}} \left( {\dot{{x}_{2}} + p_{3} x_{3} - p_{3} I_{b} - p_{2} x_{2} } \right) - p_{2} x_{1} \dot{{x}_{2}} + p_{3} x_{1} \left( { - p_{4} x_{3} + p_{4} I_{b} + u\left( t \right)} \right) + p_{5} \dot{{x}_{4}}$$

35 $$e^{ \cdots } = W\left( t \right) + p_{3} x_{1} u\left( t \right)$$

where:

$$W\left(t\right)=\ddot{{x}_{1}}\left({p}_{1}+{x}_{2}\right)+\dot{{x}_{1}}\left(\dot{{x}_{2}}+{p}_{3}{x}_{3}-{p}_{3}{I}_{b}-{p}_{2}{x}_{2}\right)-{p}_{2}{x}_{1}\dot{{x}_{2}}+{p}_{3}{p}_{4}{I}_{b}{x}_{1}-{p}_{3}{p}_{4}{x}_{1}{x}_{3}+{p}_{5}\dot{{x}_{4}}$$

Hence, we want to verify that $\dot{V}(e,\dot{e})<0$ , then from Eq. (28):

36 $$e\dot{e}+\dot{e}\ddot{e}<0$$

When we analyze Eq. (36), it can be achieved within the following four cases:

*Case 1:*

If $e$ and $\dot{e}$ are both positive sign, it requires that:

37 $$\ddot{e}<-e$$

By taking the third time derivative of Eq. (37), it yields:

38 $$\dddot e < - \dot{e}$$

By substitution the value of $\dot{e}$, $\dddot e$ from Eqs. (29), (35) respectively in Eq. (38), it yields:

39 $$W\left(t\right)+{p}_{3}{x}_{1}u\left(t\right)<\dot{{x}_{1}}$$

By Solving for $u(t)$ from Eq. (39), it yields:

40 $$u\left(t\right)<\frac{\dot{{x}_{1}}-W\left(t\right)}{{p}_{3}{x}_{1}}$$

*Case 2:*

If $e$ and $\dot{e}$ are both negative sign, it requires that:

41 $$\ddot{e}>e$$

By taking the third time derivative of Eq. (41), it yields:

42 $$\dddot e > \dot{e}$$

By substitution the value of $\dot{e}$, $\dddot e$ from Eqs. (29), (35) respectively in Eq. (42), it yields:

43 $$W\left(t\right)+{p}_{3}{x}_{1}u\left(t\right)>-\dot{{x}_{1}}$$

By Solving for $u(t)$ from Eq. (43), it yields:

44 $$u\left(t\right)>\frac{-\dot{{x}_{1}}-W\left(t\right)}{{p}_{3}{x}_{1}}$$

*Case 3:*

If $e$ is positive sign and $\dot{e}$ is a negative sign, it requires that:

45 $$\ddot{e}>-e$$

By taking the third time derivative of Eq. (45), it yields:

46 $$\dddot e > - \dot{e}$$

By substitution the value of $\dot{e}$, $\dddot e$ from Eqs. (29), (35) respectively in Eq. (46), it yields:

47 $$W\left(t\right)+{p}_{3}{x}_{1}u\left(t\right)>\dot{{x}_{1}}$$

By Solving for $u(t)$ from Eq. (47), it yields:

48 $$u\left(t\right)>\frac{\dot{{x}_{1}}-W\left(t\right)}{{p}_{3}{x}_{1}}$$

*Case 4:*

If $e$ is negative sign and $\dot{e}$ is a positive sign, it requires that:

49 $$\ddot{e}<e$$

By taking the third time derivative of Eq. (49), it yields:

50 $$\dddot e < \dot{e}$$

By substitution the value of $\dot{e}$, $\dddot e$ from Eqs. (29), (35) respectively in Eq. (50), it yields:

51 $$W\left(t\right)+{p}_{3}{x}_{1}u\left(t\right)<-\dot{{x}_{1}}$$

By Solving for $u(t)$ from Eq. (51), it yields:

52 $$u\left(t\right)<\frac{-\dot{{x}_{1}}-W\left(t\right)}{{p}_{3}{x}_{1}}$$

Consequently, the constraints in Eqs. (40), (44), (48), and (52) for the control signal $U$ ensure the asymptotic stability of the system.

## Proposed system for regulation of BGL

**Figure 12:**
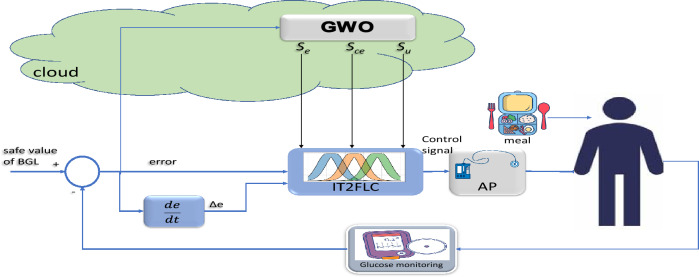
Block diagram of proposed IoMT-based IT2FLC for T1DP.

The optimized IT2F controller is proposed to regulate the BGL of T1DP. The controller is assessed using EBMM which give an accurate model for the patient. The parameters of the EBMM are different from one patient to another so, the IT2F controller is not fixed and its parameters are tuned using metaheuristic technique. GWO is used to adjust the FOU of the membership functions of IT2F controller to cope with the change in the uncertainty and perturbations. Figure 12 shows the block diagram for regulation of BGL of T1DP using interval T2FLC with GWO based on IoMT.

The inputs of the controller are the error signal $e$ and the change of error signal $\Delta e$, $u$ is the output of controller, $R$ refers to the desired BGL, and $G$ refers to the measured BGL of T1DP.

53 $$u=f\left(e,\Delta e\right)$$

54 $$e=R-G$$

**Figure 13:**
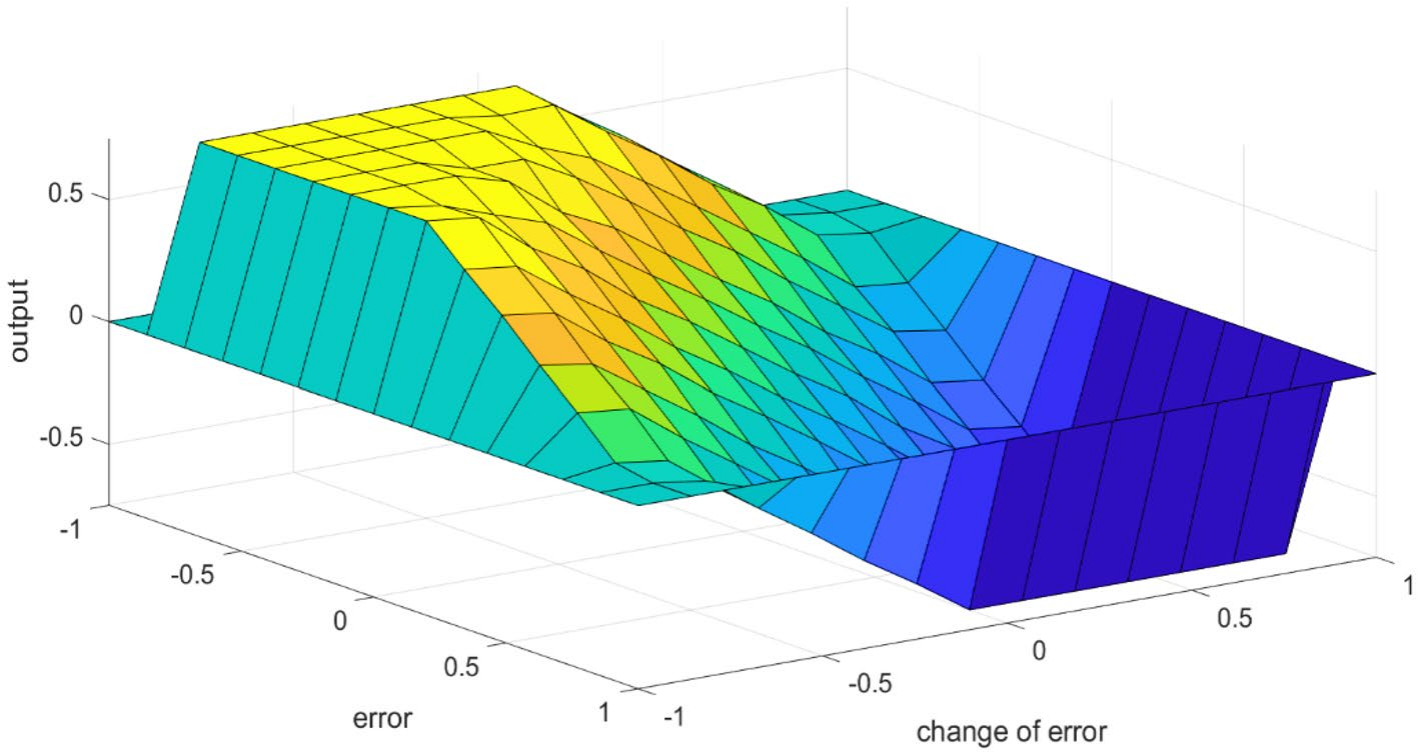
Fuzzy inference system output surface.

The surface output for inference of IT2F is shown in Fig. 13. Table 2 represents the knowledge base defining the rules for the desired relationship between the inputs and output.

**Table 2:**
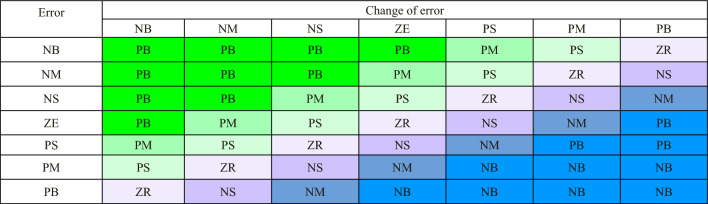
Rule base of error and change of error.

Each of the signals $e,\Delta e,u$ has its own FOU where ${s}_{e},{s}_{ce},{s}_{u}$ in Fig. 12 refers to width for FOU of interval T2MF for $e,\Delta e,u$ respectively, we will tune the FOU of each signal to adjust the controller to cope with the uncertainty of the process. In this paper ${s}_{e},{s}_{ce},{s}_{u}$ of the proposed controller are tunning GWO technique to obtain the optimal response^34^.

## Simulation results

In this section, the performance and the effectiveness of interval T2FLC in regulating BGL for EBMM of T1DP are investigated under four different scenarios using MATLAB/Simulink platform^37^. The challenges facing the proposed controller become more difficult the more we go from scenario 1 to scenario 4 to ensure its efficiency and robustness. Consequently, the main objective of the proposed controller is to lower BGL after a meal is provided to a T1DP more than 180 mg/dl from the state of hyperglycemia within 120 min to avoid post-prandial hyperglycemia, while it must preserve BGL not less than at any instance 50 mg/dl to avoid severe hypoglycemia. Also, the required.

The parameters, ${s}_{e},{s}_{ce},{s}_{u}$ of interval T2FLC are equal to 0.0080217, 0.0045828, and 0.0013535 respectively. It was obtained from GWO algorithm with the configuration of 20 iteration numbers for each of 30 search agents, and minimization of the objective function that is equal to sum of integral absolute of error signal and integral absolute of control signal.

*Scenario 1:*

In this Scenario, it is assumed that the simulation period is 800 min, the initial conditions of the state variables of extended BM model at $t=0 \mathrm{min}$ are ${x}_{1}=250 \mathrm{mg}/\mathrm{dl}$ reflects that T1DP is already in the state of hyperglycemia, ${x}_{2}=0 {\mathrm{min}}^{-1}$, ${x}_{3}=7 \mathrm{mU}/\mathrm{l}$ ensuring that there is no insulin infusion before the starting of simulation, ${x}_{4}=10 \mathrm{mg}/\mathrm{dl}/\mathrm{min}$ that means a large meal disturbance with a very high glucose content is provided at the start of the simulation. Also it is assumed that the basal values for glucose and insulin are ${G}_{b}=80 \mathrm{mg}/\mathrm{dl}$, ${I}_{b}=7 \mathrm{mU}/\mathrm{l}$ respectively and the nominal values for parameters ${p}_{1}$,${p}_{2}$, ${p}_{3}$, ${p}_{4}$, and ${p}_{5}$ of EBMM from Table1 are used.

*Scenario 2:*

Here, a 100 virtual T1DPs are used in simulation in order to investigate the behavior of interval T2FLC under both parametric uncertainty of EBMM and random initial conditions of BGL that state variable ${x}_{1}$ changes randomly from 80 to 350 mg/dl. At start of each simulation period the parameters ${p}_{1}$,${p}_{2}$, ${p}_{3}$, ${p}_{4}$, and ${p}_{5}$ are randomly chosen from Table1 to constitute a 100 virtual T1DPs. The initial conditions of the state variables of EBMM at $t=0$ are ${x}_{2}=0 {\mathrm{min}}^{-1}$, ${x}_{3}=7 \mathrm{mU}/\mathrm{l}$. Also it is assumed that T1DPs are in fasting condition that ${x}_{4}=0 \mathrm{mg}/\mathrm{dl}/\mathrm{min}$ during the whole simulation period and the basal values for glucose and insulin are ${G}_{b}=80 \mathrm{mg}/\mathrm{dl}$,${I}_{b}=7 \mathrm{mU}/\mathrm{l}$ respectively.

*Scenario 3:*

The configuration of this scenario is as for scenario 2 except that a single meal disturbance with a very high rate of glucose absorption is provided at the start of each simulation that ${x}_{4}=10 \mathrm{mg}/\mathrm{dl}/\mathrm{min}$. So the objective of this case is to investigate the behavior of interval T2FLC for each of parametric uncertainties of extended BM model, random initial condition of BGL, and meal disturbance.

*Scenario 4:*

Now, 100 different type-1 diabetic patients are tested with 1440 min running period to simulate a more realistic situation for different T1DPs. Consequently, the objective of this scenario is to investigate the behavior of the proposed controller within 24 h for different patients’ daily life. So, a three different meals 5 mg/dl/min for breakfast, 8 mg/dl/min for lunch, and 8 mg/dl/min dinner are provided to each of 100 T1DPs at 480 min (8 am), 720 min( 12 am), and 1200 min( 8 am) respectively. At start of each simulation period the parameters ${p}_{1}$,${p}_{2}$, ${p}_{3}$, ${p}_{4}$, and ${p}_{5}$ are randomly chosen from Table1 to constitute a 100 virtual T1DPs, the initial conditions of the state variables are ${x}_{2}=0 {\mathrm{min}}^{-1}$, ${x}_{3}=7 \mathrm{mU}/\mathrm{l}$, ${x}_{4}=0 \mathrm{mg}/\mathrm{dl}/\mathrm{min}$ , and the basal values for glucose and insulin are ${G}_{b}=80 \mathrm{mg}/\mathrm{dl}$,${I}_{b}=7 \mathrm{mU}/\mathrm{l}$ respectively.

## Discussions

**Figure 14:**
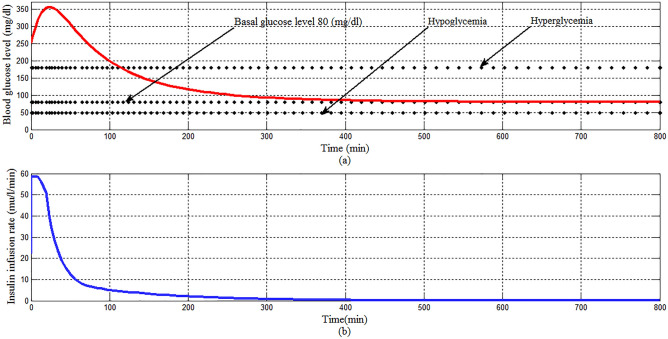
(**a**) BGL response for case 1 (**b**) insulin infusion rate for Scenario 1.

**Figure 15:**
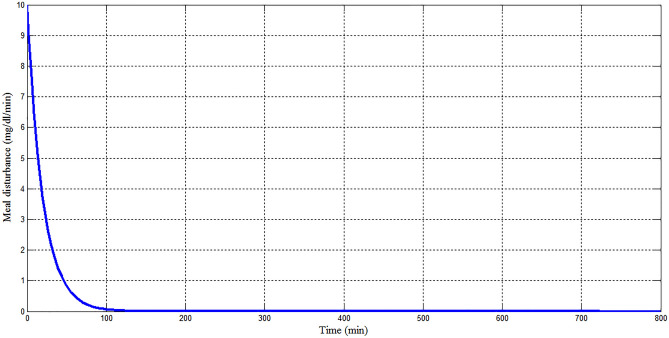
Meal disturbance for Scenario 1.

In Scenario 1: Fig. 14a showed that the proposed controller lowered BGL more than 180 mg/dl from the state of hyperglycemia within 120 min in spite of a meal disturbance with a very high glucose content was provided at the start of the simulation, the shape of the meal disturbance is shown in Fig. 15, in addition BGL stabilized at basal glucose level of 80 mg/dl without any hypoglycemia at any instance as desired, as shown in Fig. 14b response of insulin infusion rate is always positive and smooth, it reflects that the proposed controller not complicated doesn't need any excessive method or algorithm to inject glucagon or glucose to avoid hypoglycemia.

**Figure 16:**
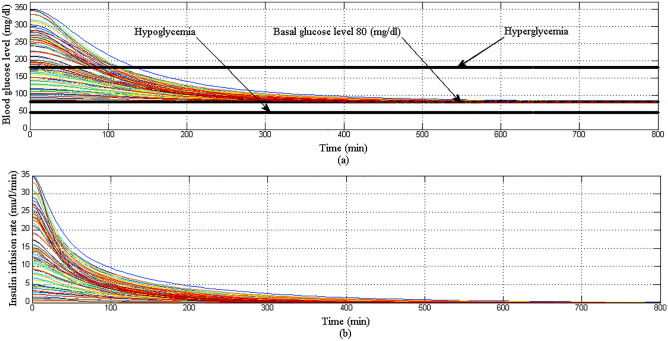
(**a**) BGL response for case 2 (**b**) insulin infusion rate for scenario 2.

In Scenario 2: with both parametric uncertainty and random initial conditions of BGL as shown in Fig. 16a the proposed controller lowered effectively BGL of all 100 virtual T1DPs more than 180 mg/dl within 120 min without any hypoglycemia at any instance. Also as shown in Fig. 16b the response of insulin infusion rate is still positive and smooth.

**Figure 17:**
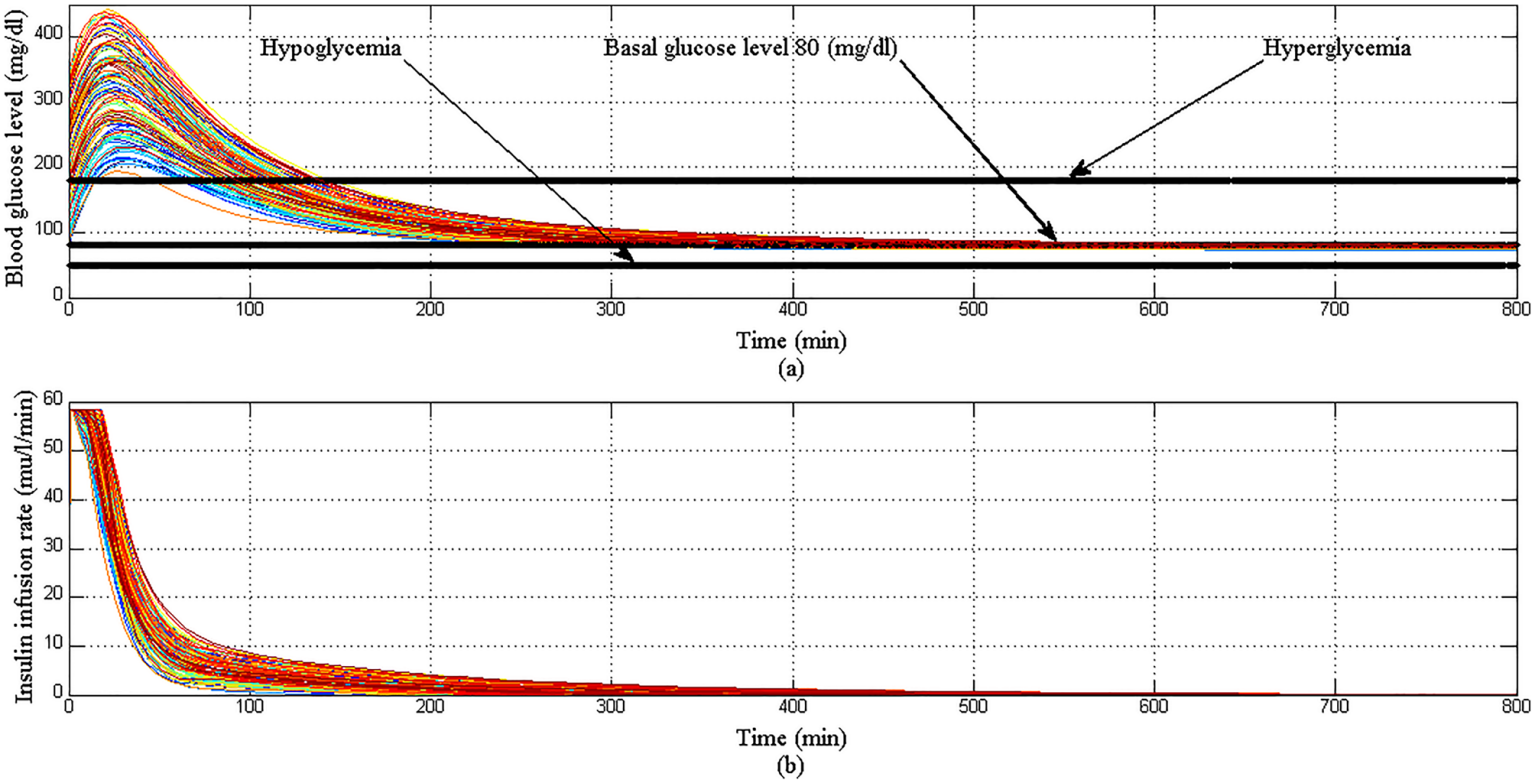
(**a**) BGL response for case 3 (**b**) insulin infusion rate for scenario 3.

In Scenario 3: despite challenges facing the proposed controller in regulating BGL became more difficult with the addition of a large meal disturbance at the start of each simulation period for each of 100 virtual T1DPs, both hyperglycemia and hypoglycemia still avoided as shown in Fig. 17a. The response of insulin infusion rate is shown in Fig. 17b.

**Figure 18:**
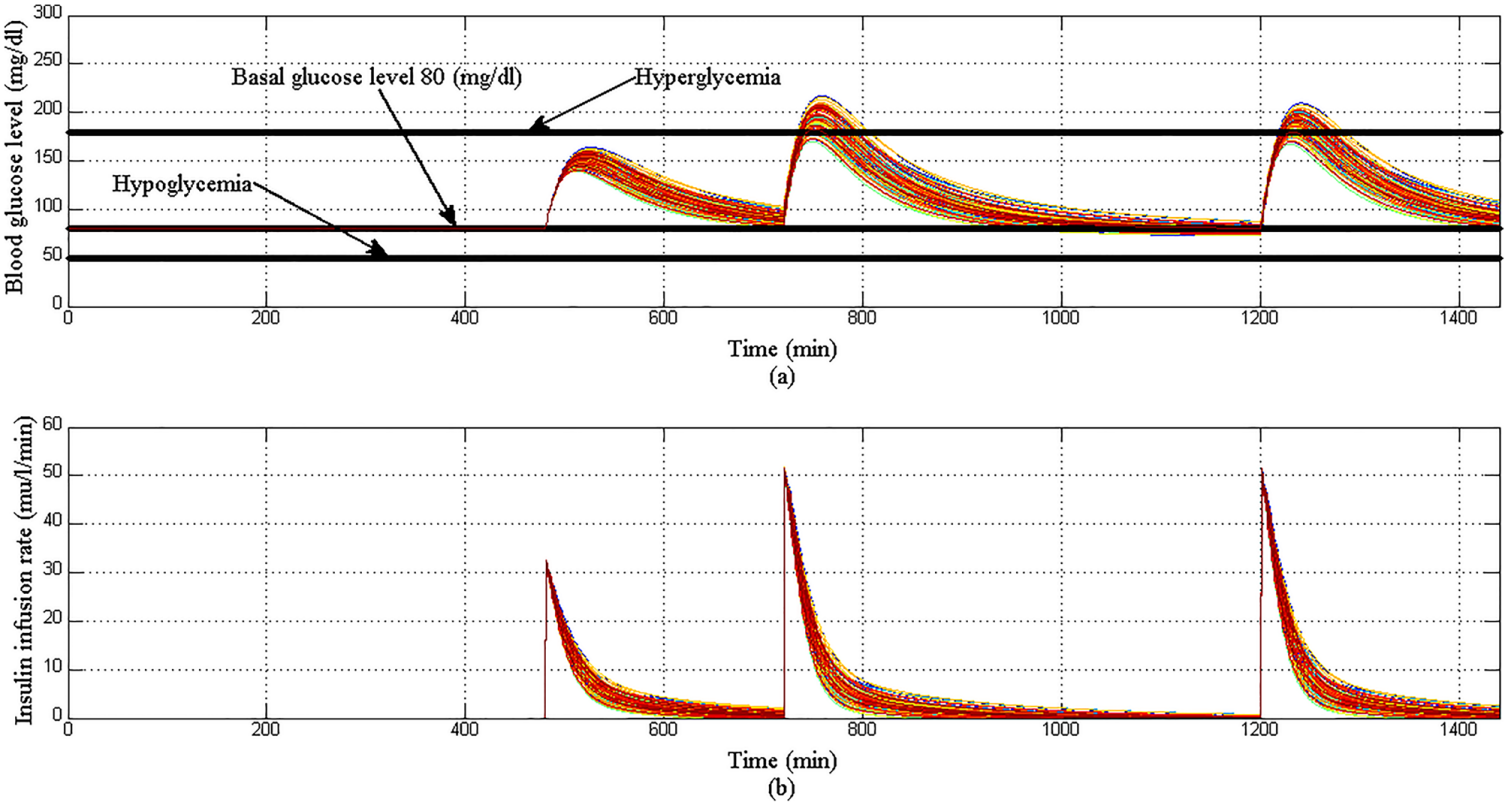
(**a**) BGL response for case 4 (**b**) insulin infusion rate for Scenario 4.

**Figure 19:**
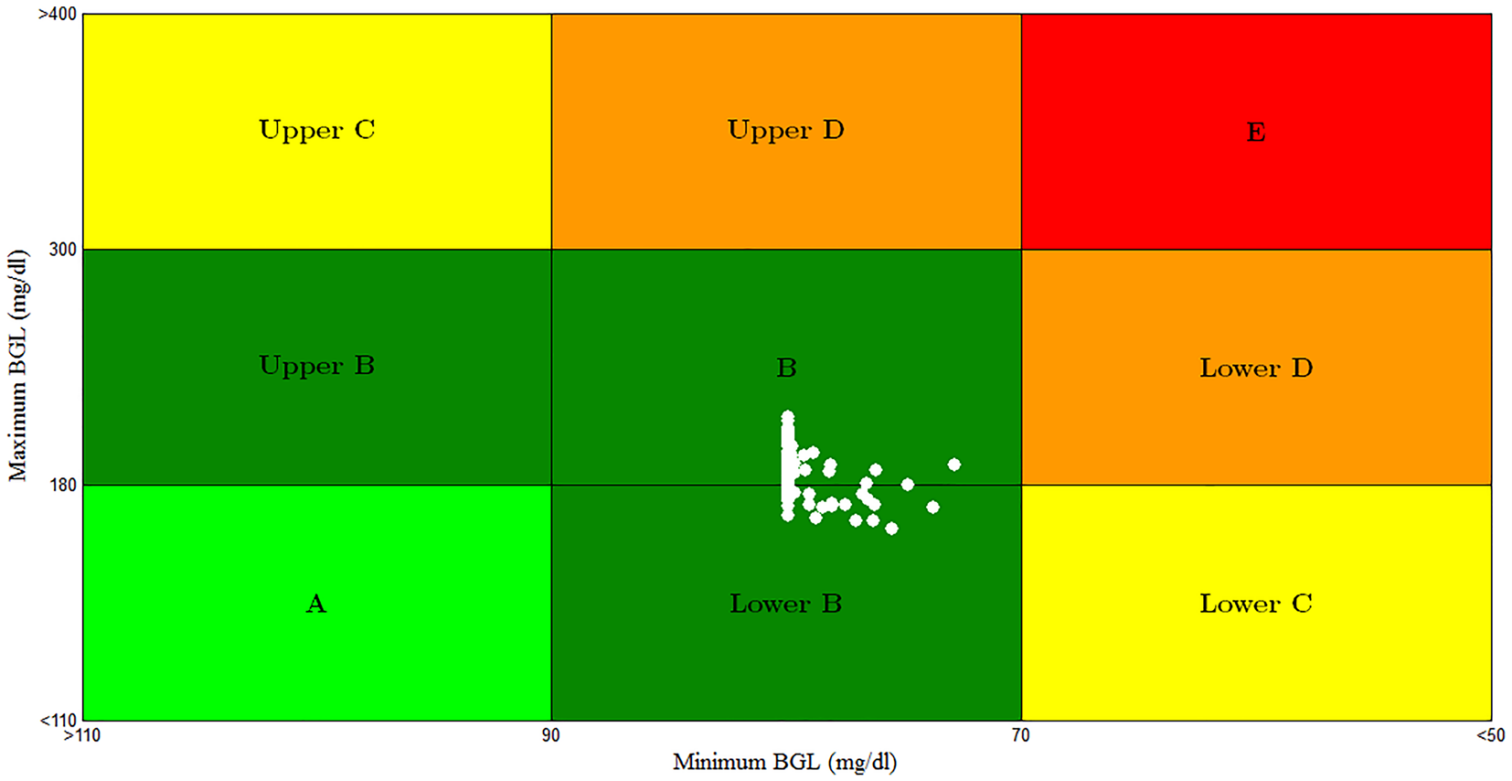
CVGA plot for Scenario 4.

In Scenario 4: as shown in Fig. 18a initially when the BGL is at basal glucose level there is no insulin infusion, but according to the amount of glucose content in each of the three meals BGL increases proportionally. Also insulin infusion rate is increased proportionally to any changes in BGL and forces it to stabilize around the basal glucose level as shown in Fig. 18b. Despites the different three meal disturbances and parametric uncertainty, both hyperglycemia and hypoglycemia were still avoided. The results of CVGA for case 4 of 100 T1DPs is shown in Fig. 19, CVGA is a graphical tool used to measure the quality of the controller in regulating the BGL for the population of T1DPs by expressing the minimum/maximum glucose values for each of T1DPs^38^, where each of T1DP in CVGA plot is expressed by a white dot, X-axis and Y-axis refers to minimum and maximum BGL within the simulation period respectively. from Fig. 19 it is noted that all 100 white dots are confined to the green safe regions grid B and grid lower B reflecting that the time period spent by 100 T1DPs in hyperglycemic state is minimized in spite of three different meals disturbance within the 24 h and the minimum glucose values for all the 100 T1DPs are between 70 and 90 mg/dl.

The model parameters that are used in the proposed technique are chosen exactly the same as in the work presented in^1^, the minimum glucose values in CVGA plot of Fig. 19 are between 70 and 90 mg/dl the same as the minimum glucose values in CVGA plot of Fig. 9 of^1^ moreover 77% of 100 T1DPs are almost centered at the basal glucose level 80 mg/dl and the rest are between 73 and 80 mg/dl. Also the maximum glucose values of Fig. 19 are between 163.5 and 210 mg/dl whereas maximum glucose value are almost between 235 and 240 mg/dl in^1^. It is considered a significant improvement in stabilizing the BGL near the basal glucose level without the occurrence of hyperglycemia or hypoglycemia situations.

From above results, it is noted that the proposed technique will stabilize and regulate the BGL at a standard-safe level. The interval T2FLC with the conjunction of GWO significantly decreases the effect of uncertainty and disturbances.

## Conclusions

A hybrid interval T2FLC with the conjunction of GWO for regulating the BGL of nonlinear extended Bergman minimal model of type 1 diabetic patient based on IoMT is proposed. The nonlinear EBMM is used to describe the dynamic behavior of glucose-insulin physiological system for T1DP under uncertainty. GWO is used to optimally tune the FOU parameters of interval T2FLC so that handle the uncertainty in the system. However, by the suggested control system, the glucose level for T1DP was regulated at the desired level. So, by changing parametric uncertainty of the model and different meals disturbance, the proposed technique is capable of stabilizing and regulating the BGL at a standard-safe level with avoiding the risks of hyperglycemia and hypoglycemia. Several scenarios are conducting for T1DP to show the superior and performance of the optimized interval T2FLC. The simulation results show that proposed controller decreases the effect of uncertainty and disturbances under different situation and with different patients. Also, the obtained injected insulin's curves are completely smooth and devoid of aberrant fluctuations, making their implementation simple. Besides, the proposed controller is conducting to different 100 type-1 diabetic patients and it observed that 77% of the samples are almost centered at the basal glucose level 80 mg/dl and the rest are also within the accepted region. As for the future work an interval T2FLC with the conjunction of other optimization techniques such as whale optimization technique will be considered for regulating BGL of T1DPs.

## Data Availability

The datasets used and/or analyzed during the current study available from the corresponding author on reasonable request.

## Abbreviations

IoMT: Internet of Medical Things
AP: Artificial Pancreas
FOU: Footprint of uncertainty
BGL: Blood glucose level
T1DP: Type-1 diabetic patient
GWO: Grey wolf optimization
T2FLC: Type 2 fuzzy logic controller
EBMM: Extended Bergman minimal model
CVGA: Control variability grid analysis
